# Cytomegalovirus Strain TB40/E Restrictions and Adaptations to Growth in ARPE-19 Epithelial Cells

**DOI:** 10.3390/microorganisms8040615

**Published:** 2020-04-24

**Authors:** Mai Vo, Alexis Aguiar, Michael A. McVoy, Laura Hertel

**Affiliations:** 1 Department of Pediatrics, University of California San Francisco, Oakland, CA 94611, USA; ngocmai1710@ucla.edu (M.V.); alexis.aguiar@ucsf.edu (A.A.); 2 Department of Pediatrics, Virginia Commonwealth University, Richmond, VA 23229, USA; michael.mcvoy@vcuhealth.org

**Keywords:** human cytomegalovirus, tropism, epithelial cells, adaptation, syncytia

## Abstract

Despite displaying broad tropism in vivo, human cytomegalovirus (CMV) contained in bodily fluids replicates inefficiently in most cultured cell types except fibroblasts. As propagation in fibroblasts leads to the accumulation of genomic changes, a number of strains were generated by serial passaging on endothelial cells. One of these, TB40/E, was shown to contain a mixture of genetically distinct virus variants, and to retain tropism for fibroblasts, endothelial and epithelial cells. Cloning of an endotheliotropic subpopulation produced the TB40-BAC4 variant, extensively used in CMV tropism studies. Because TB40-BAC4 represents only one of the different variants comprising TB40/E, we generated a series of epithelial-cell adapted stocks derived from a TB40/E mixed stock, rather than from TB40-BAC4. Within two passages on ARPE-19 cells, virus populations were produced with the ability to enter and initiate replication with similar efficiencies in both epithelial cells and fibroblasts. Although the ability to release progeny also increased, cell-free virus yields from ARPE-19 cells remained consistently two to three-logs lower than from fibroblasts, hinting at the existence of a post-entry and post-genome synthesis block in epithelial cells. Multinucleated syncytia also rapidly appeared exclusively in ARPE-19 cell cultures, where their numbers and dimensions increased with virus passage. Irrespective of the number of infected nuclei comprising each syncytium, however, only one cytoplasmic virion assembly compartment was consistently observed, leading us to speculate that improvements in entry efficiency associated with ARPE-19 cell adaptation lead to the development of syncytia, which may negatively affect progeny release by limiting the amount of resources available to maturing virions.

## 1. Introduction

The broad tropism characteristic of human cytomegalovirus (CMV) is at the source of the severe disease caused by this virus in immunocompromised individuals. Virtually all cell types except lymphocytes, neutrophils, eosinophils and basophils can support CMV replication in vivo, with fibroblasts, epithelial, endothelial, smooth muscle and myeloid cells being predominant targets of either lytic or latent infection [1]. Because of this, viral spread and organ involvement can be substantial during acute infection of patients lacking adequate immune defenses.

By contrast, and for reasons not yet fully understood, CMV shed in bodily fluids such as urine and saliva do not efficiently replicate in most cell types in vitro, except for fibroblasts [2,3]. The vast majority of CMV laboratory strains were thus historically derived by propagation of clinical isolates in this latter cell type. The realization in the 1990s that serial passaging on fibroblasts results in highly adapted strains with very restricted tropism due to the accumulation of genomic mutations and/or deletions [4,5], spurred the development of new viral strains more or less extensively propagated on endothelial cells, after isolation and minimal passaging on fibroblasts [6,7,8].

One such strain, TB40/E, has since become popular because of its ability to retain tropism for both fibroblasts and endothelial cells, even after extensive (>40 times) passaging in fibroblasts after the original propagation and plaque purification in endothelial cells [8]. While maintenance of the endotheliotropic phenotype implies a genetic origin, polymerase chain reaction (PCR) amplification and sequencing of select genomic regions revealed the presence of genetically different populations within the TB40/E strain [9], a finding initially confirmed by the identification of two viral variants, one containing an intact (TB40E-Lisa) and one containing a mutant (TB40E-Bart) copy of the *UL141* open reading frame (ORF) [10], and subsequently by restriction fragment length polymorphism analysis of nine different TB40/E bacterial artificial chromosome (BAC) clones [11]. Interestingly, only two of these clones, TB40-BAC4 and TB40-BAC12, could generate plaques in endothelial cell monolayers, suggesting that the endotheliotropic component of the TB40/E virus population is subdominant. The finding that another clone, 40E, selected from a different round of TB40/E plaque purification on endothelial cells, and its BAC derivative RV-TB40-BAC_KL7_-SE, also initiated infection in endothelial cells at rates ~7-fold lower than in fibroblasts further corroborates this notion [12].

Full genome sequencing of the TB40-BAC4 clone supported its use in viral tropism studies, which confirmed that CMV entry into epithelial cells requires the presence of both the trimeric complex (TC), comprised of the gH/gL/gO glycoproteins and necessary for entry into all cell types [13], and of the pentameric complex (PC), comprised of gH/gL plus the UL128, UL130 and UL131A proteins, and necessary for entry into endothelial cells [14,15,16,17,18,19,20,21,22] and some but not all myeloid cell types [23,24,25,26,27].

TB40-BAC4 virions released by fibroblasts were reported to contain higher amounts of TC than of PC [28]. Similar data were obtained after fibroblast infection with a GFP-expressing TB40-BAC4 derivative TB40/E*wt*-GFP [29] reconstituted and passaged once in fibroblasts [30], while virions released by fibroblasts infected with TB40/E*wt*-GFP reconstituted and passaged once in epithelial cells contained higher levels of PC than of TC [30]. These findings suggest that growth in epithelial cells may be associated with the selection of genetic variants capable of producing PC-rich(er) virions, but the mechanisms supporting these cell-dependent differences in gH/gL partitioning into each complex remain obscure. A G > T substitution in the first intron of the TB40-BAC4 *UL128* ORF was however shown to participate in limiting the PC content of virions when inserted into the genome of CMV strain Merlin [31], while the UL148 protein was shown to reduce the rate whereby newly synthesized gO is targeted for endoplasmic reticulum-associated degradation, thus supporting its incorporation into the TC [32,33]. The US16 protein was also found to promote pentamer incorporation on the envelope, potentially by interacting with UL130 in the cytoplasmic virion assembly compartment (VAC) at late times post-infection [34]. Whether these mechanisms are differentially regulated in a cell type-specific manner, however, has not been investigated, so their contributions to explaining the differences in TC/PC content of virions produced by fibroblasts vs epithelial cells remain unknown.

Despite being an outstanding tool to investigate CMV infection in a broad variety of cell types, TB40-BAC4 is a clonal strain, and hence represents only one of the different variants comprising the original TB40/E virus population. BAC insertion also resulted in the inadvertent deletion of a ~3 kb genomic fragment spanning the *IRS1* to *US1* region [12]. Like all BAC clones, production of viral stocks requires transfection of the BAC DNA in mammalian cells, a process notoriously associated with the acquisition of often undetected mutations [35], as genome sequencing of reconstituted stocks is not routinely performed.

In this work, we sought to produce a population of epithelial cell-adapted viruses derived from TB40/E, rather than from TB40-BAC4. As the TB40/E stock is presumably comprised of both PC-rich and TC-rich virions, we speculated that the TC-rich portion might prevail in stocks produced in human foreskin fibroblasts (HFF), and that the proportion of PC-rich virions could be increased by passaging in ARPE-19 cells. We show that: (1) TB40/E initiation of infection in ARPE-19 cells is highly impaired but not completely blunted, indicating that the stock still contains epitheliotropic variants; (2) the ability to enter and initiate infection in ARPE-19 cells is rapidly acquired upon passaging, suggesting that the expansion of pre-existing PC-rich variants, rather than the advent of adaptive mutations followed by selection, is likely occurring; (3) a G_754_ > T transversion at the 3′ end of the *UL128* ORF in the TB40/E stock, resulting in a STOP > Leu change in the amino acid sequence of the protein effectively extending its length by 19 residues, is reversed in the adapted stocks, presumably restoring expression of a UL128 protein with the correct size; (4) despite entering into ARPE-19 cells and HFF with similar efficiencies, cell-free yields from ARPE-19 cells infected with adapted stocks remain significantly lower compared to HFF; (5) defects in cell-free progeny production are not due to deficiencies in expression or localization of viral proteins involved in DNA replication, nor in viral DNA synthesis or intracellular progeny maturation processes; (6) infection of ARPE-19 cells by adapted stocks leads to the development of large syncytia, each containing a single VAC but multiple nuclei. As the PC is thought to be required for cell–cell fusion and syncytium formation in epithelial cells [2,36], we speculate that the substantial improvements in entry efficiency that accompany the selection of PC-rich variants are counterbalanced by the advent of complications in the process of progeny release, possibly due to the lack of sufficient cellular resources, or of plasma membrane surface, in the large infection-induced syncytia.

## 2. Materials and Methods

### 2.1. Cells

HFF (a kind gift from E. S. Mocarski, Emory University, Atlanta, GA) and ARPE-19 cells (ATCC^®^ CRL-2302™) were propagated in Dulbecco’s modified Eagle medium (DMEM) supplemented with 10% fetal clone serum III (HyClone), 100 U/mL penicillin, 100 μg/mL streptomycin, 4 mM 2-[4-(2-hydroxyethyl)piperazin-1-yl]ethanesulfonic acid (HEPES), and 1 mM sodium pyruvate (Gibco, Life Technologies, NY).

### 2.2. TB40/E Stock Adaptation in ARPE-19 Cells

CMV strain TB40/E, a kind gift from Dr. Christian Sinzger (University of Ulm, Ulm, Germany), was passaged twice on HFF (stock 31915) prior to use in infection of ARPE-19 cells in a T75 flask at a multiplicity of infection (MOI) of 10 plaque forming units (pfu)/cell. At day 11 post-infection (pi), the culture supernatant was collected (Figure S1, SA), mixed at a 1:3 ratio with fresh DMEM and transferred to a T175 flask of ARPE-19 cells. This initiated the “supernatant lineage”. Cells were also harvested by trypsinization, sonicated on ice, mixed with fresh medium and added to a T175 flask of ARPE-19 cells. This initiated the “cell lineage”.

*Supernatant lineage*. At day 5 post SA-infection, 50% of the supernatant was replaced with fresh DMEM. Five days later, the entire supernatant was collected (Figure S1, SB), mixed at a 1:1 ratio with fresh DMEM and transferred to a new T175 flask of ARPE-19 cells. Cells were also harvested, pelleted by centrifugation, mixed at a 1:1 ratio with uninfected ARPE-19 cells and plated in four T175 flasks. At day 5 post SB-infection, 50% of the supernatant was replaced with fresh DMEM. Four days later, the entire supernatant was collected and stored at −80 °C (Figure S1, SC). Cells were also collected, mixed at a 1:1 ratio with uninfected ARPE-19 cells and plated in four T175 flasks. At day 5 post-mixing of the SB cells with uninfected cells, 50% of the supernatant was replaced with fresh DMEM. Six days later, the entire supernatant was collected, stored at −80 °C (Figure S1, SD1), and replaced with fresh DMEM. At day 11 post-infection, supernatant and cells were collected and stored at −80 °C (Figure S1, SD2 and SD2 cells). At day 5 post-mixing of the SC cells with uninfected cells, 50% of the supernatant was replaced with fresh DMEM. Five days later, the entire supernatant was collected, stored at −80 °C (Figure S1, SE1), and replaced with fresh DMEM. At day 13 post-infection, supernatant and cells were collected and stored at −80 °C (Figure S1, SE2 and SE2 cells).

*Cell lineage*. At day 5 post-infection with the cell sonicate, 50% of the supernatant was replaced with fresh DMEM. Four days later, the supernatant was collected (Figure S1, CB), part of it was mixed at a 1:1 ratio with fresh DMEM and transferred to a new T175 flask of ARPE-19 cells. Cells were also harvested, mixed at a 1:1 ratio with uninfected ARPE-19 cells and plated into four T175 flasks. At day 4 post CB infection, the entire supernatant was collected and stored at −80 °C (Figure S1, CC). Cells were also collected, mixed at a 1:1 ratio with uninfected ARPE-19 cells and plated into four T175 flasks. At day 4 post-mixing of the CB cells with uninfected cells, the entire supernatant was collected, stored at −80 °C (Figure S1, CD1), and replaced with fresh DMEM. At day 7 post-infection, supernatant and cells were collected and stored at −80 °C (Figure S1, SD2 and SD2 cells). At day 6 post-mixing of the CC cells with uninfected cells, 50% of the supernatant was replaced with fresh DMEM. Four days later, the entire supernatant was collected, stored at −80 °C (Figure S1, CE1), and replaced with fresh DMEM. At day 13 post-infection, supernatant and cells were collected and stored at −80 °C (Figure S1, CE2 and CE2 cells).

### 2.3. Immunofluorescence Staining Analyses (IFA)

Infected cells in HFF and ARPE-19 populations were detected by immunofluorescence staining analyses (IFA) as previously described [26]. Briefly, cells grown on 12 mm diameter glass coverslips were fixed in 1% formaldehyde for 30 min, permeabilized in 0.5 % Triton-X 100 for 20 min, blocked in 20% fetal bovine serum for 30 min and incubated with a mouse monoclonal antibody directed against the viral immediate-early (IE) proteins 1 and 2 (MAb810, 1:600, EMD Millipore, Temecula, CA & MAB810X-AF488, 1:100x, EMD Millipore, Temecula, CA), UL44 (pp52 P12021, 1:100, Virusys, Taneytown, MD), UL57 (CMV ICP8 P1209, 1:100, Virusys, Taneytown, MD), or UL99 (CMV pp28 P1207, 1:500, Virusys, Taneytown, MD) for one hour at room temperature (RT), followed by Alexa-Fluor 488- or Alexa-Fluor 594-conjugated secondary antibodies (1:200, Invitrogen, Carlsbad, CA, United States, and Jackson Immunoresearch, West Grove, PA, United States) for another hour. Nuclei were labeled with Hoechst 33342 (0.2 mg/mL; Molecular Probes, Eugene, OR, United States) for three min. Samples were viewed using a Nikon Eclipse E600 fluorescence microscope equipped with Ocular imaging software.

### 2.4. Virus Titrations

Titers of culture supernatants and cell-associated virus, obtained by sonication of cell pellets for ~3 s with a Branson Ultrasonics Sonifier 150, were determined by staining HFF with MAb810 at 24 h post-infection.

### 2.5. Polymerase Chain Reaction (PCR) Amplifications, Sequencing and Sequence Alignments

PCR amplification of the *UL128-131A* genomic region was performed using primers 5′-CAATATCGCCATCTCTATCG-3′ (forward) and 5′-CTTTCGGTTCCAACTCTTTCC-3′ (reverse) and the following cycling conditions: 98 °C for 1 min, 40 cycles of 98 °C for 10 sec, 59 °C for 30 sec, 72 °C for 45 sec, and 72°C for 10 min. Sanger sequencing of PCR products was performed by Quintara Biosciences (Hayward, CA) using primers: 5′- GATAAACACCACTATCGC-3′ and 5′-ACTTACACCTTCTGCACC-3′ (*UL128*), 5′-AACTGACCGCCTCGGAAATG-3′ and 5′-GACGAAGCAGAAGCCGTAGC-3′ (*UL131A*), and 5′-GCCCGGAGCCTCGAGTTCAGCG-3′ (*UL130*). Sequence alignments were performed using the CLC Sequence Viewer Version 7.7.1 software with a gap open cost of 5, a gap extension cost of 5, and a free gap end cost. The *UL128*, *UL130* and *UL131A* sequences were obtained from the following NCBI human betaherpesvirus 5 complete or partial genome sequence files: TB40/E Lisa (KF297339), RV-TB40-BAC_KL7_-SE (MF871618), TB40-BAC4 (EF999921), TB40-E_UNC (KX544839), TB40/E (AY446866), UxCA_Merck_UNC (KX544840) and HANRTR6 (KY490075).

### 2.6. Real-Time Quantitative Genomic PCR

Genomic DNA was extracted from infected and uninfected cells using the OmniGenX PureSpin gDNA mini prep kit (E&K Scientific, Santa Clara, CA, USA). Real-time, quantitative PCR reactions were performed as described [27,37] using the iTaq SYBR green supermix with ROX (Bio-Rad, Hercules, CA, USA) and an ABI7900 thermocycler (Applied Biosystems, Carlsbad, CA, USA), with primers hybridizing to exon 2 of the viral *UL122* and *UL123* ORFs (forward primer: 5′-GGCCGAAGAATCCTCAAAA-3′ and reverse primer: 5′-TCGTTGCAATCCTCGGTCA-3′). The following cycling parameters were used: 95 °C for 2 min to activate the iTaq polymerase, followed by 40 cycles of template denaturation at 95 °C for 15 s, primer annealing at 51 °C (*UL122/123*) or 57 °C (*albumin*) for 30 s, and product extension at 72 °C for 30 s. Absolute quantifications of viral and cellular genome amounts were obtained using a standard curve made by serial dilutions of plasmid pON303. The number of viral genome copies per cell was calculated as number of viral DNA copies/number of IE+ cells per reaction.

### 2.7. Statistical Analysis

The distributions of any two data sets were compared using the Kolmogorov-Smirnov or the Mann-Whitney test using GraphPad Prism 8.3.1. Differences were considered significant at *p* < 0.05.

## 3. Results

### 3.1. TB40/E Grown of Human Foreskin Fibroblasts (HFF) Shows Poor Tropism for ARPE-19 Epithelial Cells

**Figure 1:**
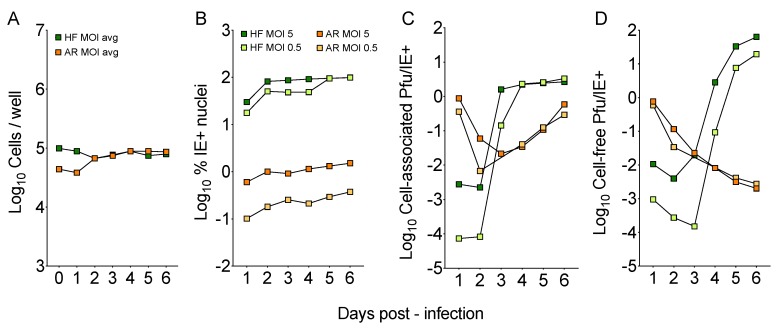
TB40/E stock tropism for fibroblasts and epithelial cells. Human foreskin fibroblasts (HFF) and ARPE-19 cells were exposed to TB40/E stock 31915 at a multiplicity of infection (MOI) of 0.5 or 5 pfu/cell. At each time post-infection, cells and supernatants were collected and used to determine the total number of cells/well (**A**), the proportion of cells expressing the nuclear IE1/IE2 proteins (**B**), the amounts of cell-associated progeny per IE+ nucleus (**C**) and the amounts of cell-free progeny per IE+ nucleus (**D**). All titrations were conducted on HFF.

To evaluate the ability of the original TB40/E stock (coded 31915) to grow on epithelial cells, HFF and ARPE-19 cells were infected at a calculated MOI of 5 or 0.5 pfu/cell (titers determined on HFF), and the number of cells/well, the proportion of IE+ nuclei, and the amount of cell-associated and cell-free virus present in each culture from day 1 to 6 pi were determined (Figure 1). While cell densities became virtually identical after day 2 (Figure 1A), the average proportion of IE+ HFF remained >200-fold (MOI = 0.5) and >80-fold (MOI = 5) higher than that in ARPE-19 cultures, which also showed a 4-fold difference in the number of antigen-positive nuclei detected at each MOI (Figure 1B). The proportions of infected HFF observed at day 1 (~40% at MOI of 0.5; ~85% at MOI of 5) were also consistent with Poisson distribution predictions for these MOIs, while proportions observed for infected ARPE-19 cells were dramatically lower (~0.2% and ~1%, respectively).

While peak progeny production was reached at day 4 pi in HFF cultures (Figure 1C, green squares), and was followed by the release of ~13 pfu (MOI of 0.5) and ~50 pfu (MOI of 5) per IE+ nucleus at day 6 pi (Figure 1D, green squares), assembly of cell-associated virus proceeded at a much slower pace in ARPE-19 cell populations (Figure 1C), and was not followed by the release of cell-free progeny (Figure 1D), so that extracellular yields per IE+ nucleus at day 6 were ~ 4-logs higher in HFF than in ARPE-19 cells.

These data suggested that, compared to HFF, TB40/E stock 31915 infection initiation in ARPE-19 cells was impaired, progression was delayed, and release of virions in the extracellular environment was compromised. The fact that entry was still occurring and that viral replication was not completely blunted, however, indicated that subpopulations of virions with epithelial cell tropism were still present within the TB40/E stock.

### 3.2. The Ability to Initiate Infection in ARPE-19 Cells is Rapidly Gained upon Passaging on Epithelial Cells

To improve TB40/E’s tropism for epithelial cells, ARPE-19 cells were infected with the 31915 stock at an MOI of 10 pfu/cell, and at day 11 pi, when plaques were visible and enlarged, both cell-free (culture supernatant) and cell-associated (cell sonicate) virus progenies were transferred to new ARPE-19 cells to initiate the “supernatant lineage” and the “cell lineage”, respectively. Passaging of each lineage was then repeated twice (Figure S1).

The efficiency of infection initiation of virus populations harvested at each passage was then measured by determining the proportion of IE+ nuclei present in HFF and in ARPE-19 cultures at day 1 post-addition of equal amounts of supernatant or of cell sonicate to each cell type (Table S1). Percentages were then used to calculate the IE+ in HFF/IE+ in ARPE-19 ratio (H/A ratio).

As expected, progeny virus released in the supernatant of ARPE-19 cells infected with the 31915 stock showed poor tropism for epithelial cells (H/A: 6.5). Two passages were however sufficient to virtually equalize the proportion of IE+ nuclei found in both cell types (SB to SE2 mean H/A values: 1.6 ± 0.6 and CB to CE2 mean H/A values: 1.8 ± 0.7), suggesting that variants competent for entry into epithelial cells and already present within the original TB40/E population were rapidly expanded, irrespective of whether the initial inoculum consisted of cell-free (supernatant lineage) or cell-associated (cell lineage) virions.

### 3.3. Efficient Entry into ARPE-19 Cells is Associated with the Selection of TB40/E Variants Carrying a TGA (STOP) Codon at the 3′ End of UL128

The *UL128-131A* genomic region was PCR amplified from the TB40/E 31915, SA, SD2, SE2, CB, CD2 and CE2 stock genomes and the Sanger sequences of the *UL128*, *UL130* and *UL131A* ORFs were aligned to those found in TB40/E (partial sequence, AY446866), TB40E-Lisa, RV-TB40-BAC_KL7_-SE and two TB40-BAC4 genome sequences (TB40-BAC4 and TB40-E_UNC), and those found in two other CMV strains, UxCA, derived from urine virus and passaged exclusively in ARPE-19 cells [2], and HANRTR6, sequenced directly from the vitreous body fluid of a kidney transplant recipient with retinitis [38].

**Figure 2:**
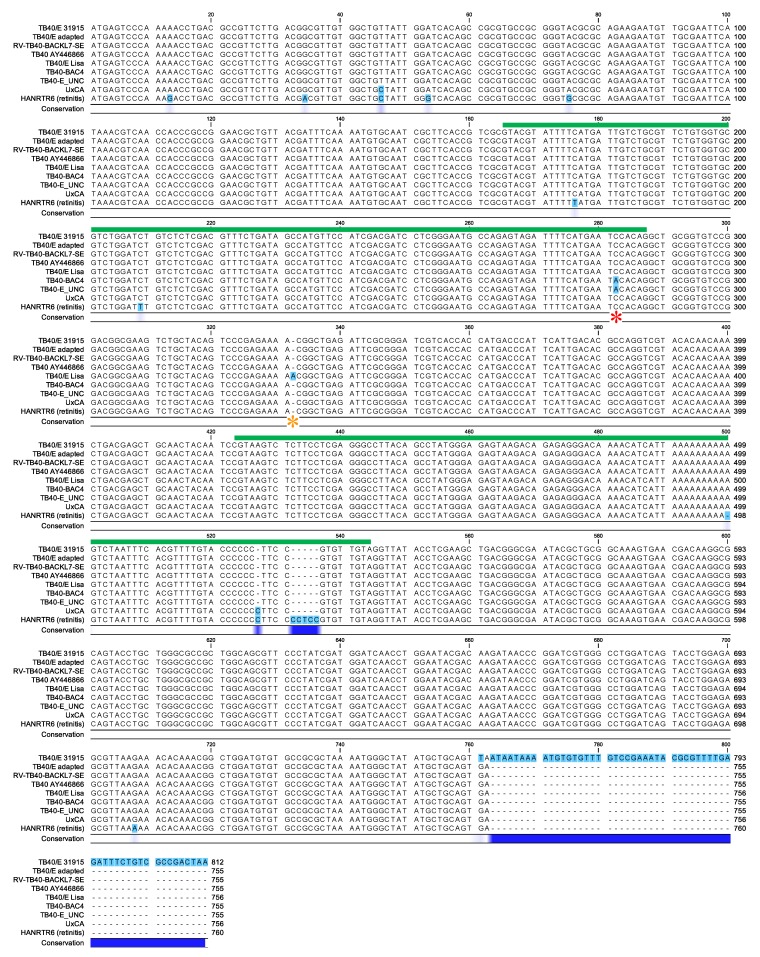
*UL128* nucleotide sequence alignment. The *UL128-131A* region in TB40/E 31915 and in the SA, SD2, SE2, CB, CD2 and CE2 stock genomes was amplified by polymerase chain reaction (PCR) prior to Sanger sequencing of the *UL128* open reading frame (ORF). All adapted strains shared the same *UL128* sequence and are thus shown as “TB40/E adapted”. Sequences were aligned to those found in the listed strains using the CLC Sequence Viewer software. Different residues are highlighted in turquoise. The degree of conservation amongst the nine sequences is shown by the intensity of the blue color in the Conservation ribbon below the alignment. The green lines denote the ORF introns. The red asterisk marks the C_282_ > A transversion exclusive to the TB40-BAC4 strain. The orange asterisk marks the A_332_ insertion exclusive to the TB40E-Lisa strain.

*UL128.* All ARPE-19 passaged stocks shared the same *UL128* nucleotide (nt) sequence, which was identical to that found in RV-TB40-BAC_KL7_-SE and in TB40/E AY446866, but diverged from those in the rest of the strains/stocks at the sites shown in Figure 2 and listed in Table S2. While the dominant *UL128* sequence in TB40/E 31915 contained a G_754_ > T transversion converting the TGA_753-5_ (STOP) codon into a Leu-coding triplet, this change was not observed in the adapted stocks. The dominant *UL128* sequence in TB40/E 31915 and in the adapted viruses also lacked the C_282_>A transversion observed in TB40-BAC4 and TB40-E_UNC, and previously associated with a 50-fold increase in cell-free virus production in HFF due to reduced splicing efficiencies of the *UL128* mRNA when transferred into the genome of CMV strain Merlin [31], or the A_332_ insertion observed in TB40E-Lisa and in TB40-BAC1, and previously reported to reduce the length of the UL128 protein by 99 amino-acids [11]. The *UL128* sequence in adapted stocks was also more similar to that in UxCA (one nt change and one insertion) than in HANRTR6 (eight nt changes and three insertions), with most of these differences being intronic or leading to amino acid changes in the protein’s signal peptide.

*UL130.* No differences were observed in the *UL130* nt sequence found in TB40/E 31915 and in each passaged stock (Table S2), with the *UL130* sequence in the adapted stocks being more similar to that in UxCA (5 changes) than that in HANRTR6 (13 changes). Interestingly, one of the changes observed in UxCA and four of those observed in HANRTR6 led to non-conservative replacements in the protein’s amino acid sequence.

*UL131A.* All passaged stocks and TB40/E 31915 shared the same *UL131A* nt sequence, which was also identical to that in UxCA except for a single synonymous mutation (Table S2). Again, several differences were observed in HANRTR6 (eight nt changes and one insertion), but all were synonymous or intronic.

In summary, TB40/E 31915 adaptation to grow in ARPE-19 cells was associated with the selection of variants devoid of the STOP_175_ > L mutation, and thus presumably containing functional PC on their virion surface due to the expression of a UL128 protein of the correct size. The predicted amino acid sequences of all three proteins as encoded by the adapted strains were also highly similar to those encoded by UxCA and HANRTR6, with the majority of non-conservative amino acid changes occurring in UL130.

### 3.4. Cell-Free Progeny Release from ARPE-19 Cells Infected with Adapted Viruses Remains Hampered

**Figure 3:**
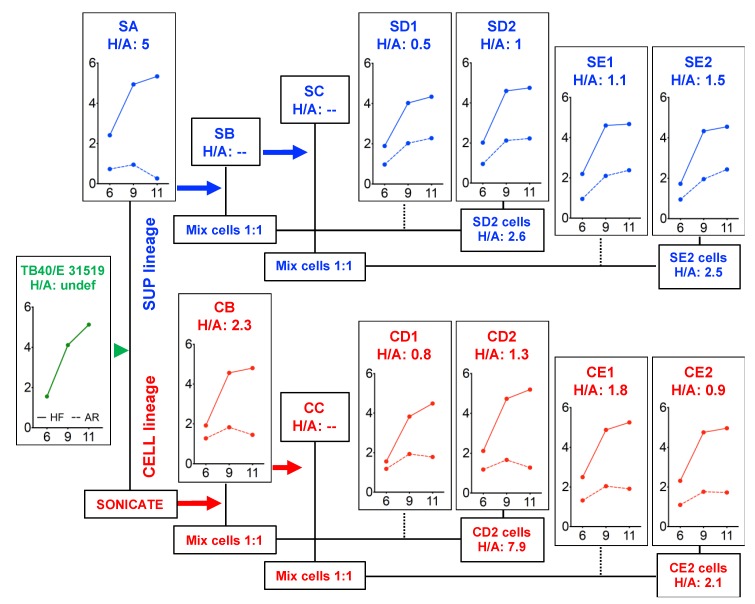
Growth properties of the parental TB40/E stock virus and of derived adapted populations. The adaptation scheme shown is the same as depicted in Figure S1. The % IE+ HFF / % IE+ ARPE-19 cell ratios (H/A) at day 1 post-infection of HFF and ARPE-19 cells infected with each stock at an MOI of 0.01 pfu/cell and the virus content of supernatants collected at days 6, 9 and 11 pi (X axis) as determined by titration on HFF (Log_10_ pfu/well, Y axis) are shown. Solid lines = virus yields from infected HFF. Dotted lines = virus yields from infected ARPE-19 cells. Green font = initial TB40/E 31519 stock. Blue font = “supernatant lineage” stocks. Red font = “cell lineage” stocks. Undef = as no IE+ cells were observed in ARPE-19 cells, the denominator of the H/A ratio is zero.

We next evaluated the ability of passaged viruses to complete their replication cycle in HFF and ARPE-19 cells infected at an MOI of 0.01 pfu/cell with cell-free virus from each stock. The number of IE+ nuclei present in each well at day 1 pi, the corresponding H/A ratios (Table S3), and progeny amounts released in culture supernatants at day 6, 9 and 11 pi were determined (Figure 3).

No IE+ nuclei, and no progeny release were observed upon infection of ARPE-19 cells with the 31915 stock, while robust growth was detected on HFF (Figure 3, TB40/E panel). As expected, the efficiency of infection initiation of the SA stock was still higher in HFF than in ARPE19 cells (H/A: 5), and although detectable cell-free progeny started to be released by ARPE-19 cells, amounts remained 50- to 100.000-fold lower than in HFF, with a marked downward trend from days 9 to 11 (Figure 3, SA panel).

Akin to what was observed in cultures exposed to equal amounts of virus, the proportions of IE+ HFF and ARPE-19 cells detected after infection at an MOI of 0.01 were almost identical (mean H/A values: 1.3 ± 0.5, Table S3). Despite this, cell-free virus production in ARPE-19 cells remained at levels 200- to 2000-fold lower than those of HFF. In addition, while virus production from epithelial cell cultures infected with supernatant lineage stocks displayed a slight upward trend from days 9 to 11 (Figure 3, SD1, SD2, SE1 and SE2 panels), no such increase was observed with cell lineage stocks (Figure 3, CB, CD1, CD2, CE1 and CE2 panels), leading to a statistically significant difference in median yields at day 11 pi (4-fold, *p* = 0.016, Mann–Whitney test, Figure S2).

Together, these data suggest that TB40/E passaging on epithelial cells leads to the rapid selection of subpopulations of viruses capable of entering into ARPE-19 cells and HFF with similar efficiencies, but not of producing comparable amounts of cell-free progeny in both cell types.

### 3.5. Viral Genome Replication and Cell-Associated Progeny Production are Similarly Efficient in ARPE-19 Cells and HFF Infected with Adapted Viruses

**Figure 4:**
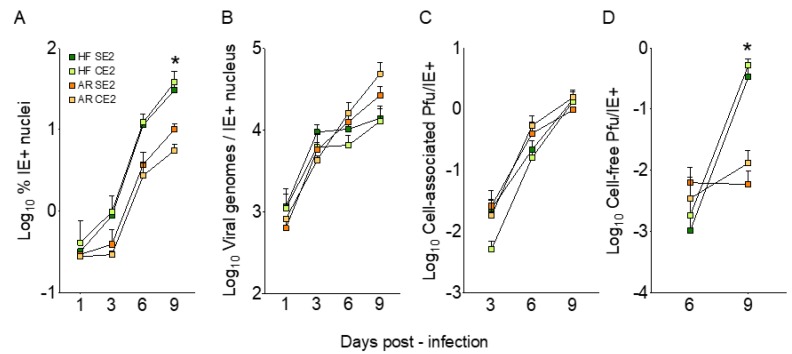
Replication of the SE2 and CE2 virus stocks in fibroblasts and epithelial cells. HFF and ARPE-19 cells were infected with each stock at an MOI of 0.01 pfu/cell and the (**A**) Proportion of nuclei expressing the IE1/IE2 proteins, (**B**) Number of viral genome copies per IE+ nucleus, (**C**) Amounts of cell-associated virus per IE+ nucleus, and (**D**) Amounts of cell-free virus per IE+ nucleus were determined. No cell-associated virus was detected at day 1, and no cell-free virus was detected at days 1 and 3. Graphs show mean ± standard deviation values from two (**B**) or three (**A**,**C**,**D**) independent experiments. * indicate statistically significant differences among samples.

To further investigate the source of the restriction affecting cell-free progeny production in epithelial cells, HFF and ARPE-19 cells were infected at an MOI of 0.01 pfu/cell with cell-free virus from the SE2 (supernatant lineage) and CE2 (cell lineage) stocks. The proportion of IE+ nuclei, the number of viral genomes per IE+ nucleus and the amount of cell-associated and cell-free virus produced from days 1 to 9 pi were then determined (Figure 4). As expected, the proportions of infected ARPE-19 and HFF at day 1 were indistinguishable, and both increased with similar kinetics from days 3 to 6 pi (~ 12-fold) and from days 6 to 9 pi (~ 3-fold), but not from days 1 to 3. Because of this initial lag, the percentages of infected ARPE-19 cells at day 9 were significantly lower (10-fold, *p* = 0.016, Mann–Whitney test) than those of HFF (Figure 4A).

By contrast, no difference was observed in the number of viral genome copies per IE+ cell in both cultures, although increases in viral DNA amounts were less prominent in HFF than in ARPE-19 cells from day 3 onward (Figure 4B); 69 ± 2 % of IE+ ARPE-19 nuclei and 60 ± 2 % of IE+ HFF also expressed the viral single-stranded DNA-binding protein UL57 at day 3 pi, and 83 ± 5 % of IE+ ARPE-19 nuclei and 64 ± 6 % of IE+ HFF also expressed the viral DNA-polymerase processivity factor UL44 at day 6 pi (not shown). Additionally, all of the UL57 signal and the vast majority of the UL44 signal (67 ± 7 % in ARPE-19 cells and 64 ± 7 % in HFF) were found in well-formed replication compartments (not shown), as expected due to the essential role of these proteins in viral DNA replication [39]. No differences were also detected in the amounts of cell-associated virus produced per IE+ nucleus by either cell type (Figure 4C), while once again, the amounts of cell-free progeny released by ARPE-19 cells at day 9 were significantly lower (60-fold, *p* = 0.016, Mann–Whitney test) than those released by HFF, irrespective of the virus stock used (Figure 4D).

Thus, while viral DNA synthesis and intracellular progeny maturation appear to proceed with similar efficiencies in both cell types, release of cell-free virions was impaired in ARPE-19 cells.

### 3.6. ARPE-19 Cultures Infected with Adapted Viruses Contain Numerous Large Syncytia at Late Times Post-Infection

**Figure 5:**
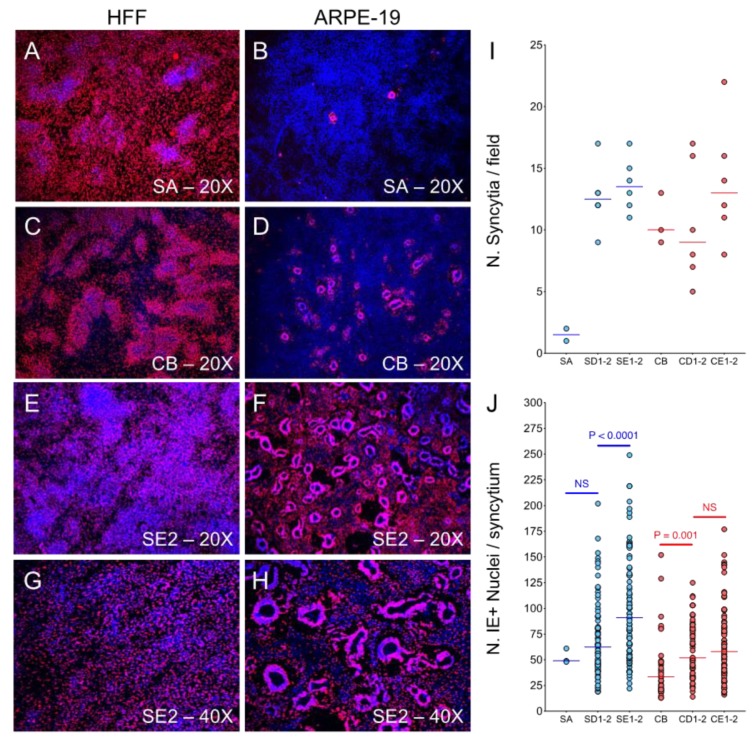
Development of syncytia in infected ARPE-19 cultures. HFF and ARPE-19 populations were infected at an MOI of 0.01 pfu/cell, fixed and stained at day 11 pi for IE. (**A**–**H**) Micrographs of infected populations stained with an anti-IE antibody (red) and with Hoechst 33342 to visualize nuclei (blue). Signal overlap appears in purple. Images were taken at a 20× or 40× magnification. (**I**) Number of syncytia and (**J**) number of IE+ nuclei per syncytium detected in 3 different, randomly selected fields of ARPE-19 cells infected with virus stocks listed on the X axis. Bars correspond to median values; *p* values correspond to Mann–Whitney comparison tests. NS = not significant.

Staining for IE expression of HFF and ARPE-19 cells infected with each stock at an MOI of 0.01 pfu/cell for 11 days revealed that virtually all HFF contained a single IE+ nucleus, irrespective of the virus used as inoculum (Figure 5A,C,E,G). By contrast, significantly lower numbers of ARPE-19 nuclei were IE+ after infection with passage one stocks (SA and CB), and the large majority of these were included in relatively small syncytia, more numerous in CB- (269/well) than in SA-infected (18/well) populations (Figure 5B,D).

Very interestingly, while the number of syncytia observed in cultures infected with cell lineage stocks remained fairly constant (Figure 5I, red circles), a sharp increase was observed in cells infected with supernatant lineage stocks after passage one (Figure 5I, blue circles). The number of nuclei included in each syncytium also appeared to progressively increase irrespective of the lineage (Figure 5J), with the largest ones, containing up to 250 nuclei, being observed in ARPE-19 cells infected with the supernatant lineage stocks SE1–SE2 (Figure 5F–H and J). No or very small syncytia were detected in infected HFF populations.

### 3.7. The Majority of Syncytia Contain Multiple IE+, UL44+ and UL57+ Nuclei but a Single UL99+ VAC

To evaluate if the viral replication cycle was still proceeding in these large syncytia, ARPE-19 cells infected with the SE2 and CE2 viruses at an MOI of 0.01 pfu/cell were stained for the IE proteins, as well as for pUL57 and pUL44, involved in viral DNA replication, and for pUL99 (pp28), involved in virion maturation and exclusively expressed after the onset of viral DNA synthesis.

**Figure 6:**
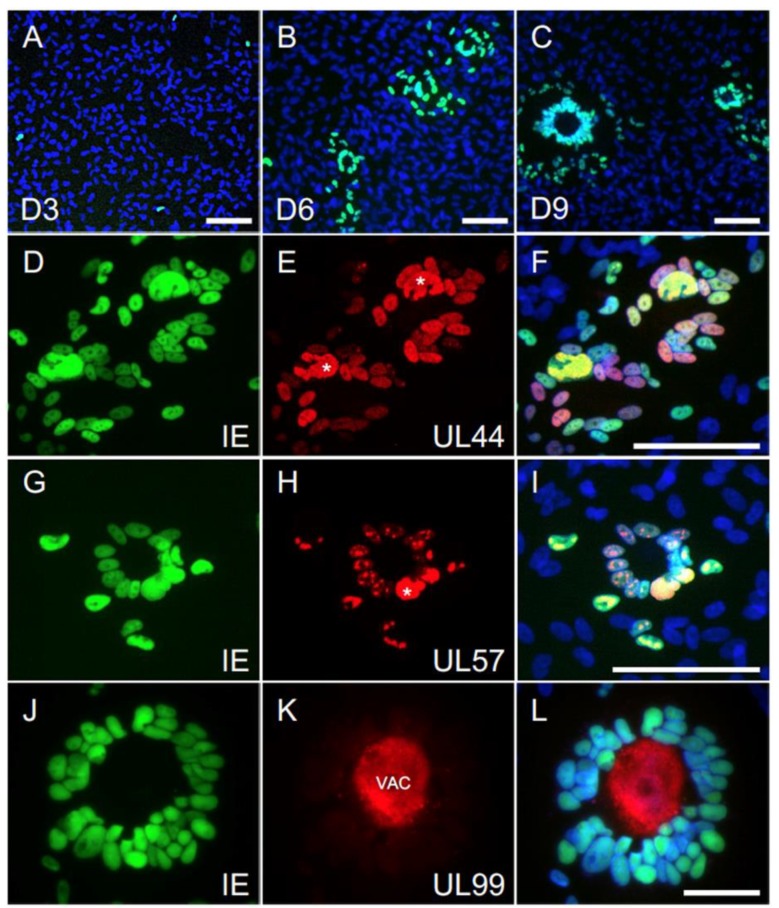
Viral antigen expression in syncytia. ARPE-19 cells were infected at an MOI of 0.01 pfu/cell with the SE2 or CE2 stocks prior to staining for IE, UL44, UL57 or UL99. (**A**–**C**) Micrographs of infected populations stained for IE (green) and with Hoechst 33342 (blue) at days 3, 6 and 9 pi (D3, D6 and D9). (**D**–**F**) Micrographs of infected populations stained for IE (green) and for UL44 (red) at day 6 pi. (**G**–**I**) Micrographs of infected populations stained for IE (green) and for UL57 (red) at day 6 pi. (**J**–**L**) Micrographs of infected populations stained for IE (green) and for UL99 (red) at day 9 pi. Merged images (**F**,**I**,**L**) also show nuclear staining with Hoechst 33342. Asterisks mark the viral DNA replication compartments. VAC = virion assembly compartment. White bars = 100 μm.

Only single IE+ cells were detectable at day 3 pi. By contrast, well-formed syncytia containing 3–20 nuclei were readily visible at day 6 in both SE2- and CE2-infected cultures, and both their initial external size (~57 μm) and nuclei content increased by ~30% and ~18%, respectively, at day 9 (Figure 6A–C and Table S4). Virtually all nuclei within each syncytium were IE+, UL44+ and UL57+ at day 6, with at least one nucleus per syncytium displaying a prominent accumulation of these proteins in very large DNA replication compartments (Figure 6D–I, asterisks). All syncytia also contained a single UL99+ VAC (Figure 6J–L), whose initial size at day 6 (~ 25 μm) was increased by ~ 13% at day 9 (Table S4). The VAC was invariably located at the center of each syncytium, surrounded by a “halo” of viral antigen+ nuclei.

Together, these results confirm that viral genome replication is not blocked in infected ARPE-19 cultures, and the presence of a sizable VAC within each syncytium further suggests that virion maturation is proceeding and leading to the production of infectious progeny. Thus, we believe that the drop in cell-free yields observed in ARPE-19 cultures is likely due to defects in progeny release, rather than assembly, and we speculate that these may be caused by a shortage of available plasma membrane surface for virion egress due to the formation of large syncytia.

## 4. Discussion

The TB40/E strain has retained a broader tropism than most passaged strains despite the use of fibroblasts for its isolation, initial propagation and subsequent amplification after plaque purification in endothelial cells. This unusual property has been proposed to depend on the presence of more than one genotypically distinct variants within the strain [9,10], some of which are strictly limited to growth on fibroblasts, and some capable of efficiently infecting additional cell types [11]. Because this genetic heterogeneity hindered the use of TB40/E in genotype–phenotype analyses, a single endotheliotropic variant was cloned (TB40-BAC4) [11], and widely used in CMV tropism studies.

While mixed stocks are not practical for the functional characterization of viral genes, they more closely mimic the behavior of clinical isolates, and may thus be better suited to investigate CMV entry and replication into tissue-derived specimens containing mixtures of cells, such as those comprising the oronasal and genital mucosae. In addition, mixed stocks may allow for a more rapid identification of tropism determinants via the selection of pre-existing variants, instead of by de novo mutagenesis.

Here, we sought to produce virus stocks with similar degrees of tropism for fibroblasts and epithelial cells, and chose TB40/E, rather than TB40-BAC4, as starting material in order to maintain a broader genetic heterogeneity.

As the endotheliotropic variants comprising the TB40/E strain appeared to be minoritarian [11], we expected the parental 31519 stock to be poorly epitheliotropic. Indeed, the proportion of IE+ cells was dramatically lower in ARPE-19 than in HFF cultures (Figure 1), but the fact that it remained detectable implied that PC-rich variants were still present within the stock and could potentially be enriched. Cell-associated progeny production was also proceeding in ARPE-19 cells, but with delayed kinetics and reduced intensities compared to HFF. At the same time (days 2–3 pi) when major bursts in progeny production were observed in HFF, virus amounts were dropping in ARPE-19 cells, showing a milder and more gradual surge only at later times pi. Possible reasons for this might be due to differences in the in the number of virions reaching the cytoplasm of HFF versus ARPE-19 cells, as well as in the kinetics and efficiency of nuclear delivery of viral genomes, all of which, in turn, may lead to disparities in the amount of viral DNA available for initiation of replication. Although infectious progeny was clearly produced by ARPE-19 cells, cell-free yields failed to rise, revealing the existence of a major block in progeny release whose nature is currently under investigation.

Despite the limited amounts of cell-free virus released from ARPE-19 cells, a “supernatant lineage” was successfully established after inoculation at high MOI (10 pfu/cell). Stocks with balanced entry tropisms for HFF and ARPE-19 cells could also be rapidly produced (Table S1), supporting the hypothesis of selection and amplification of PC-rich variants already present within the starting TB40/E population, as opposed to the generation of new variants via spontaneous mutation.

Sanger sequencing of PCR amplicons spanning the *UL128-UL131A* region revealed the existence of a single nt change (G_754_ > T) in the *UL128* ORF of TB40/E 31519 (Figure 2), converting the ORF’s STOP codon into a Leu-coding triplet and effectively extending the UL128 amino acid sequence by 19 residues. To our knowledge, this mutation has not been previously reported, and is the second one, after the E_72_ > STOP change detected in the TB40-Lisa [10] and TB40-BAC1 [11] strains, to result in the (potential) disruption of UL128 protein function. We also confirmed that the C_282_ > A intronic mutation reported to reduce the extent of *UL128* expression [31] is exclusive to TB40-BAC4, while the C_620_ > G mutation, resulting in a C_207_ > S amino acid change possibly modifying the function of UL130 [9] is exclusive to TB40/E AY446866 (Table S2).

Together, these data reinforce the notion that the TB40/E strain comprises several co-existing variants, some expressing a functional UL128 protein and capable of entry into epithelial and endothelial cells, and some expressing truncated or extended versions of UL128, and more adapted to grow in fibroblasts.

A total of 35 unique nt changes were also detected in the *UL128*, *UL130* and *UL131A* ORFs as found in TB40/E versus UxCA and HANRTR6. Eight of these changes resulted in amino acid substitutions in UL130, the most polymorphic of the three ORFs, and in the signal peptide of UL128. As the UxCA strain was never cultured on fibroblasts, and the HANRTR6 was directly sequenced from clinical material, these changes may be significant and may participate in modulating CMV tropism by altering UL128′s cytoplasmic levels or UL130′s function. Several additional viral ORFs aside from PC components have been reported to modulate CMV tropism for specific cell types (e.g., *UL148*, *US16*, *RL13*, *UL116* etc.) [32,40,41,42,43]. We thus expect that the ongoing whole genome sequencing of TB40/E 31915 and adapted stocks will reveal additional changes of potential relevance.

Despite the rapid equalization of entry efficiencies in HFF and ARPE-19 cells, the gap in cell-free yields remained wide, with ARPE-19 cells releasing ~200- to 1700-fold less virus than HFF at peak times (Figure 3, Figure 4 and Figure S2). This was not associated with impairments in viral DNA synthesis, or in the expression and localization of viral proteins involved in DNA replication, or in the efficiency of intracellular progeny production (Figure 4 and Figure 6 and Table S4). Rather, any defect(s) reducing cell-associated yields in ARPE-19 cells infected with TB40/E 31519 appeared to be corrected by adaptation (compare Figure 1C to Figure 4C), leaving release of cell-free virus as the only severely impaired step in the viral replication cycle.

Because infection by adapted viruses led to the formation of numerous large syncytia containing a single central VAC, we speculate that reductions in the extent of membrane surface available for virion egress, and/or shortages in cellular resources required for proper VAC functioning may contribute to the reduction of cell-free viral yields. Although the emergence of syncytia in CMV-infected cultures has been reported since the 1970s [44,45,46,47], and multinucleated giant cells have been observed in the organs of severely infected individuals [48,49,50,51,52] and in the retina of patients with CMV retinitis [53] the mechanisms involved in their formation, and their significance for viral fitness have not been thoroughly evaluated.

Several CMV-encoded mediators and enhancers of cell-cell fusion events have been identified [2,36,54,55,56,57,58,59,60,61,62], and the appearance of syncytia has been consistently linked to increases in the PC content of virions. Repair of the *UL131A* ORF, for instance, endowed the PC-negative AD169 strain with the ability to enter epithelial cells, but also led to the formation of syncytia after high MOI infection of both epithelial and fibroblast cultures [63]. Similar results were obtained after fibroblast infection with TS15-rN, a Towne strain carrying a repaired version of the *UL130* ORF, compared to cells infected with the parental PC-negative strain TS15 [2]. Finally, syncytia formation was also inhibited in infected epithelial and fibroblast cultures incubated with antibodies against PC components [2,36]. Thus, we surmise that the selection and amplification of PC-rich variants following TB40/E 31519 adaptation is directly linked to the emergence of the observed syncytiogenic behavior.

In contrast to data collected with the BADrUL131 and the TS15-rN strains (AD169 and Towne background, respectively) [2,22,63], syncytia were not observed in HFF monolayers, suggesting that epithelial cells may be more prone to membrane fusion events than fibroblasts, or that additional nt changes present in AD169 but not in the TB40/E genome contribute to this process. Syncytia formation in ARPE-19 cultures also occurred exclusively at late times post-infection, indicating that the de novo synthesis of viral glycoproteins is likely required. Moreover, the number of syncytia per field was higher, and the MOI required to induce their appearance much lower than reported for VR1814-infected cells [24,36], suggesting that fusion events likely occurred between infected and uninfected cells, rather than between adjacent infected cells.

Finally, syncytia formation may result from the selection and amplification of variants capable of directing higher levels of fusogenic proteins/complexes to the plasma membrane, hence supporting the direct transmission of virions or capsids between fused cells. This may be advantageous to viral spread by protecting virions from neutralization by extracellular antibodies.

The predominance of syncytia over single cells observed at late times post-infection was, however, associated with substantial losses in total viral yields. Although our data do not provide direct evidence that syncytia formation and low cell-free yields are causally related, the presence of a single VAC and reduced plasma membrane surfaces typical of fused cells may severely restrict the quantity and availability of cellular resources needed to produce and release infectious virions. Lower overall yields may thus represent the trade-off for an immunologically covert mode of cell-to-cell transmission.

## Supplementary Materials

The following are available online at https://www.mdpi.com/2076-2607/8/4/615/s1, Figure S1. Schematic diagram of TB40/E’s serial passage on epithelial cells as described in Materials and Methods; Table S1. Percentage of IE+ cells present in culture at day 1 post-infection of HFF or ARPE-19 cells with equal amounts (500 ul) of each virus population; Table S2. Nucleotide changes in the *UL128*, *UL130* and *UL131A* ORFs found in each of the listed strains as compared to adapted stocks; Table S3. Number of IE+ cells/well present in HFF or ARPE-19 cultures at day 3 post-infection at an MOI of 0.01; Figure S2. HFF and ARPE-19 were infected with supernatant lineage (SD1-SE2) or cell lineage (CB-CE2) stocks at an MOI of 0.01 pfu/cell; Table S4. Characteristics of syncytia present at days 6 and 9 post-infection with the SE2 and CE2 viruses.
