# Supplementary material for: Cytomegalovirus Strain TB40/E Restrictions and Adaptations to Growth in ARPE-19 Epithelial Cells
Source: Microorganisms. 2020 Apr 24;8(4):615. doi: 10.3390/microorganisms8040615 (PMC7232150; doi:10.3390/microorganisms8040615)
Supplement: Supplementary file 1 [file microorganisms-08-00615-s001.pdf]

microorganisms

Article

Cytomegalovirus strain TB40/E restrictions and adaptations to growth in ARPE-19 epithelial cells

Mai Vo, Alexis Aguiar, Michael A. McVoy and Laura Hertel

Supplementary Data

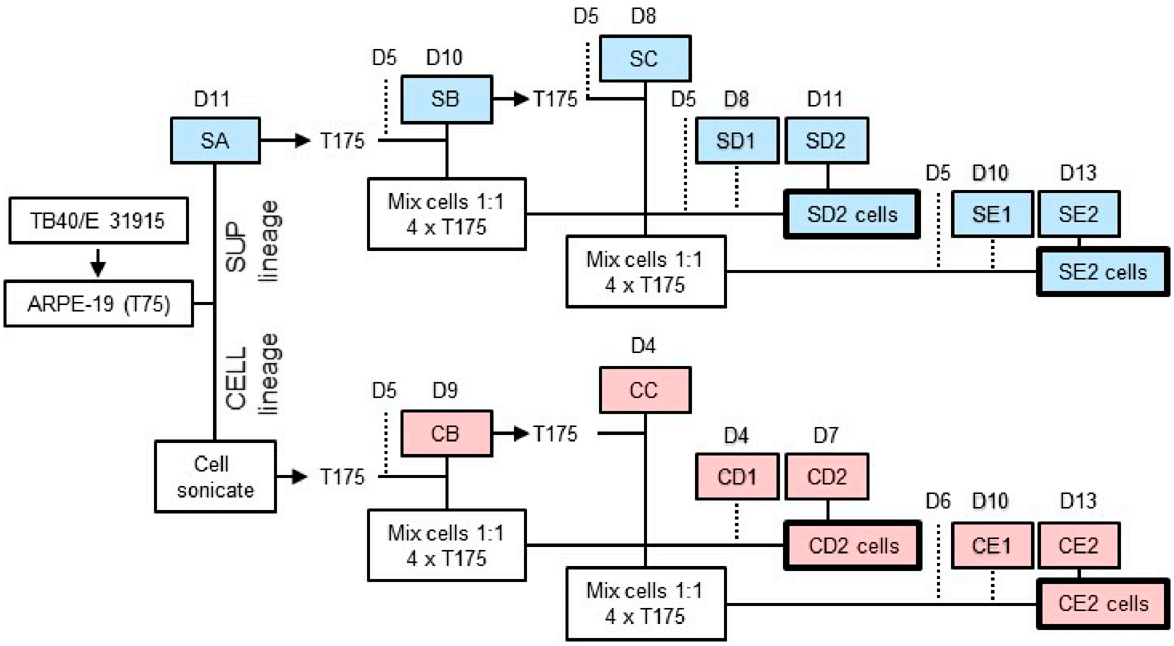

Figure S1. Schematic diagram illustrating serial passage of TB40/E on epithelial cells as described in Materials and Methods. ARPE-19 cells in a T75 flask were infected with TB40/E stock 31915 at an MOI of 10 pfu/cell. At day 11 pi, both cell-free (culture supernatant) and cell-associated (cell sonicate) virus progenies were transferred to new ARPE-19 cells in separate T175 flasks to initiate the "supernatant lineage" (blue boxes) and the "cell lineage" (red boxes). A portion of the original supernatant (SA stock) was stored for subsequent infection and titration analyses, while the entire cell sonicate was used to initiate the cell lineage (consequently, there is no CA stock). Both lineages were serially passaged twice. Colored boxes connected by vertical lines represent supernatant stocks (SA to SE2 and CB to CE2). Colored boxes with a thicker border represent cell pellet stocks (SD2 cells, SE2 cells, CD2 cells and CE2 cells). Dotted lines indicate media changes. D = day.

Table S1. Percentage of IE⁺ cells present in culture at day one post-infection of HFF or ARPE-19 cells with equal amounts (500 ul) of each virus population.

Stock	*Lineage*	*% IE⁺ HFF*	*% IE⁺ ARPE-19*	*H/A ratio*
SA	Supernatant	0.91	0.14	6.5
SB	Supernatant	0.18	0.07	2.4
SC	Supernatant	0.05	0.02	2.3
SD1	Supernatant	1.50	1.37	1.1
SD2	Supernatant	2.47	1.70	1.5
SE1	Supernatant	4.16	3.75	1.1
SE2	Supernatant	40.91	34.03	1.2
CB	Cell	1.05	0.95	1.1
CC	Cell	0.05	0.02	2.5
CD1	Cell	0.70	0.66	1.1
CD2	Cell	5.84	2.00	2.9
CE1	Cell	3.65	2.34	1.6
CE2	Cell	28.14	16.67	1.7
SD2 cell*	Supernatant	6.20	2.41	2.6
SE2 cell*	Supernatant	9.49	3.75	2.5
CD2 cell*	Cell	13.14	1.65	2.9
CE2 cell*	Cell	11.19	5.28	2.1

* 100-fold dilution of stock virus

Table S2. Nucleotide changes in the *UL128*, *UL130* and *UL131A* ORFs found in each of the listed strains as compared to adapted stocks. Percentages in the Outcome column report the Sneath's dissimilarity index value between the two replaced amino acids (Sneath, P.H. Relations between chemical structure and biological activity in peptides. J Theor Biol 1966, 12, 157-195, doi:10.1016/0022-5193(66)90112-30). Ins = insertion. Underlined text highlights changes occurring in more than one strain/stock.

ORF	*Strain/Stock*	*Nt change*	*Aa change*	*Outcome*
UL128	TB40/E 31915	G_{754} > T	$STOP_{175}$ > L	UL128 extension - 19 aa
UL128	TB40E-Lisa	Ins A_{332}	E_{72} > STOP	UL128 truncation - 99 aa
UL128	TB40-BAC4	C_{282} > A	–	Reduced splicing efficiency
UL128	TB40-E_UNC	C_{282} > A	–	Reduced splicing efficiency
UL128	RV-TB40-BAC_{KL7}-SE	None	–	–
UL128	UxCA	<u>T_{46} > C</u>	–	–
		<u>Ins C_{526}</u>	–	Intron
UL128	HANRTR6 (retinitis)	A_{13} > G	N_5 > D	14 %; Signal peptide
		G_{34} > A	A_{12} > T	20 %; Signal peptide
		<u>T_{46} > C</u>	–	None
		A_{53} > G	D_{18} > T	29 %; Signal peptide
		A_{75} > G	–	–
		C_{176} > T	–	Intron
		C_{209} > T	–	Intron
		Ins A_{499}	–	Intron
		<u>Ins C_{526}</u>	–	Intron
		Ins $CCTCC_{530-34}$	–	Intron
		G_{707} > A	–	–
UL130	TB40/E (AY446866)	C_{620} > G	C_{207} > S	23 %
UL130	UxCA	<u>A_{87} > G</u>	–	–
		<u>A_{178} > C</u>	<u>L_{60} > I</u>	5 %
		<u>T_{233} > C</u>	<u>L_{78} > S</u>	23 %
		C_{279} > T	–	–
		<u>A_{390} > G</u>	–	–
UL130	HANRTR6 (retinitis)	A_6 > G	–	–
		<u>A_{87} > G</u>	–	–
		G_{99} > A	–	–
		T_{119} > C	L_{40} > P	23 %
		<u>A_{178} > C</u>	<u>L_{60} > I</u>	5 %
		G_{227}> A	R_{76} > Q	23 %
		<u>T_{233} > C</u>	<u>L_{78} > S</u>	23 %
		C_{339} > T	–	–
		G_{380} > A	R_{127} > Q	23%
		<u>A_{390} > G</u>	–	–
		A_{501} > G	–	–
		T_{540} > C	–	–
		C_{621} > T	–	–
UL131A	UxCA	T_{477} > C	–	–
UL131A	HANRTR6 (retinitis)	G_{69} > A	–	–
		A_{75} > G	–	–
		A_{243} > G	–	Intron
		T_{259} > C	–	Intron
		GC_{295-6} > CT	–	Intron

	$G_{298} > A$	–	Intron
	$G_{314} > A$	–	Intron
	$C_{327} > T$	–	Intron
	$G_{453} > A$	–	–

Table S3. Number of IE+ cells/well present in HFF or ARPE-19 cultures at day 3 post-infection at an MOI of 0.01.

Stock	*Lineage*	*N. IE+ HFF*	*N. IE+ ARPE-19*	*H/A ratio*
TB40/E	Initial stock	444.2	0.0	–
SA	Supernatant	377.4	76.0	5.0
SB	Supernatant	–	–	–
SC	Supernatant	–	–	–
SD1	Supernatant	414.9	786.9	0.5
SD2	Supernatant	360.4	350.2	1.0
SE1	Supernatant	629.4	554.3	1.1
SE2	Supernatant	599.8	411.6	1.5
CB	Cell	810.3	353.4	2.3
CC	Cell	–	–	–
CD1	Cell	511.9	653.9	0.8
CD2	Cell	545.1	412.2	1.3
CE1	Cell	1070.7	593.5	1.8
CE2	Cell	461.3	496.6	0.9

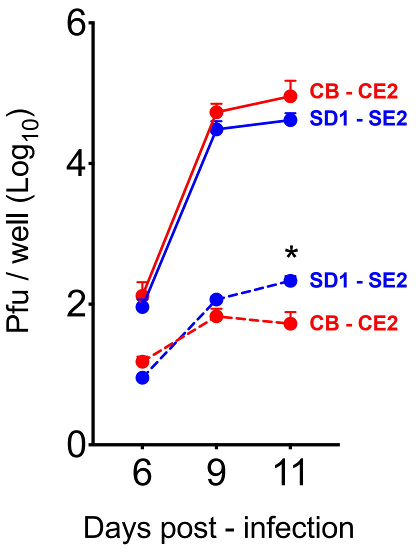

Figure S2. HFF (solid lines) and ARPE-19 cells (dashed lines) were infected with supernatant lineage (SD1-SE2) or cell lineage (CB-CE2) stocks at an MOI of 0.01 pfu/cell. At the indicated days pi, supernatants were collected and titered on HFF. Median and median absolute deviation values are shown. The asterisk marks statistically significant differences between sample median values (P = 0.016, Mann Whitney test).

Table S4. Characteristics of syncytia present at day six and nine post-infection with the SE2 and CE2 viruses. Twenty to 40 individual syncytia were evaluated for each parameter in three independent experiments. Mean and standard deviation are shown.

	SE2 day 6	***SE2 day 9***	***CE2 day 6***	***CE2 day 9***
External ∅ (µm)	60 ± 17	78 ± 18	55 ± 13	79 ± 22
Nuclei / syncytium	10 ± 4	21 ± 12	10 ± 4	24 ± 20
IE^+/ syncytium	100 ± 0	98 ± 3	99 ± 2	99 ± 2
UL44+ / syncytium	95 ± 10	ND	100 ± 0	ND
UL57+ / syncytium	99 ± 4	ND	99 ± 5	ND
VAC ∅ (µm)	25 ± 7	33 ± 9	23 ± 6	32 ± 10
